# 

**Published:** 1891-05

**Authors:** 


Vol XII.] *PUBLISHED MONTHLY AT THREE DOLLARS A YEAR.*
*SINGLE COPIES, THIRTY CENTS.* [No. 5.

LIBRARY
Indexed
S. G. O.

### THE JOURNAL

OF

### COMPARATIVE MEDICINE

AND

### VETERINARY ARCHIVES.

EDITED BY

W. A. CONKLIN, Ph. D., D. V. S.,

*Director of Zoological Gardens, New York.*

RUSH SHIPPEN HUIDEKOPER, M. D., Veterinarian, (*Alfort.*)

*Professor of Sanitary Medicine and Veterinary Jurisprudence,*

*American Veterinary College,*

*New York.*

MAY, 1891.

WILLIAM R. JENKINS, Publisher,

New York.

LONDON: BALLIERE, TINDALL & COX.

